# Blood Transfusions and Tumor Biopsy May Increase HCC Recurrence Rates after Liver Transplantation

**DOI:** 10.1155/2017/9731095

**Published:** 2017-01-05

**Authors:** Daniel Seehofer, Robert Öllinger, Timm Denecke, Moritz Schmelzle, Andreas Andreou, Eckart Schott, Johann Pratschke

**Affiliations:** ^1^Department of General, Visceral and Transplantation Surgery, Charité Campus Virchow, Berlin, Germany; ^2^Department of Radiology, Charité Campus Virchow, Berlin, Germany; ^3^Department of Gastroenterology and Hepatology, Charité Campus Virchow, Berlin, Germany

## Abstract

*Introduction.* Beneath tumor grading and vascular invasion, nontumor related risk factors for HCC recurrence after liver transplantation (LT) have been postulated. Potential factors were analyzed in a large single center experience.* Material and Methods.* This retrospective analysis included 336 consecutive patients transplanted for HCC. The following factors were analyzed stratified for vascular invasion: immunosuppression, rejection therapy, underlying liver disease, age, gender, blood transfusions, tumor biopsy, caval replacement, waiting time, Child Pugh status, and postoperative complications. Variables with a potential prognostic impact were included in a multivariate analysis.* Results.* The 5- and 10-year patient survival rates were 70 and 54%. The overall 5-year recurrence rate was 48% with vascular invasion compared to 10% without (*p* < 0.001). Univariate analysis stratified for vascular invasion revealed age over 60, pretransplant tumor biopsy, and the application of blood transfusions as significant risk factors for tumor recurrence. Blood transfusions remained the only significant risk factor in the multivariate analysis. Recurrence occurred earlier and more frequently in correlation with the number of applied transfusions.* Conclusion.* Tumor related risk factors are most important and can be influenced by patient selection. However, it might be helpful to consider nontumor related risk factors, identified in the present study for further optimization of the perioperative management.

## 1. Introduction

Hepatocellular carcinoma (HCC) is a common indication for either liver resection or transplantation, depending on the patient's preserved liver function [1]. However, in HCCs with vascular invasion, tumor recurrence is a frequent cause of death after both resection and transplantation [2–5]. In contrast, especially in the absence of vascular invasion the risk of tumor recurrence is low after liver transplantation (LT) [2]. Therefore patient selection for LT is inter alia based on surrogate parameters for vascular invasion like the Milan criteria [6]. Using adequate patient selection 5-year survival rates above 70% are reported after LT [7, 8].

Although tumor-specific factors, namely, grading and vascular invasion, are the most relevant predictors of recurrence [2, 9], other nontumor related parameters might be important for the development of HCC recurrence. For example, an association between operative blood loss and HCC recurrence rate has been described after liver resection [10]. Also the type and intensity of immunosuppression are supposed to influence recurrence rates [11]. Purpose of the present study was to analyze potential risk factors for HCC recurrence on long-term outcome after LT.

## 2. Materials and Methods

A total number of 336 consecutive patients, transplanted with known or incidental hepatocellular carcinoma (HCC) in cirrhosis, were retrospectively analyzed from 1989 to 2008. Before listing macroscopic vascular invasion and extrahepatic disease were excluded. In case of lymph node involvement LT was not performed.

Since 1997 bridging efforts during waiting time were only applied sporadically; however since 2003 bridging was performed on a regular basis. In case, mainly TACE using doxorubicin, cisplatin, and iodized oil was applied. Additional bridging strategies comprised liver resection, radio frequency ablation (RFA), and in some cases brachytherapy. Surgical technique of LT experienced slight changes over the years. Before 2000 caval replacement was performed, since 2000 in most patients a modified piggy-back technique was applied.

Cyclosporine or tacrolimus was widely used as standard immunosuppressive agents. Since 1992 tacrolimus with tapered steroids was employed in most cases. In the early phase an induction therapy with ATG or IL-2 receptor antibodies was performed. Azathioprine or mycophenolate-mofetil was additionally used if required due to rejection episodes or renal impairment. Between 2001 and 2006 sirolimus was used in some patients, mainly in those with more advanced tumor stages, based on meeting of the cost for off-label use by the responsible health insurance. Since 2006 a total of 57 HCC patients were enrolled in a prospective randomized multicenter trial primarily using sirolimus for long-term immunosuppression [12].

All patients were followed up in our outpatient clinic on a regular basis. Alpha-fetoprotein (AFP) serum levels as well as abdominal ultrasound, chest X-ray, and CT scans were performed every 6–12 months. HCC recurrence was defined as detection of new lesions with typical HCC appearance. Suspect lesions were subsequently confirmed or disproved by biopsy.

Data of are given as mean and standard error (SEM). Comparisons of categorical and continuous variables were performed using the chi-square test and the Man-Whitney *U*-test, respectively. Patient survival and recurrence rates were calculated using the Kaplan Meier method. Tumor recurrence was counted as an event, whereas death from other reasons was censored. All nontumor related risk factors were tested for their prognostic significance by using a stratified log rank test. Stratification by a variable with known prognostic value such as vascular invasion or tumor stage increases the power and protects against baseline imbalances. Likewise patients were divided into two strata according to the most dominant tumor related risk factor vascular invasion (yes/no). The stratified log rank test analyses the respective tested variable over both strata, resulting in one *p* value. Nontumor related variables with a significant prognostic impact on univariate analysis were included in a multivariate analysis applying the Cox multiple stepwise regression model. All differences were considered statistically significant if the *p* value was less than 0.05. All statistical analyses were performed using PASW Statistics 18.0 (SPSS Inc., Chicago, IL, USA).

## 3. Results

The overall 1-, 5-, and 10-year patient survival rates were 90%, 70%, and 54%, respectively. For further analysis of nontumor related factors, the patient population was divided into one subgroup with low risk of tumor recurrence, that is, tumors without microvascular invasion (group: no vascular invasion, *n* = 222) and one subgroup with high risk of tumor recurrence (vascular invasion, *n* = 114). Patients with vascular invasion had significantly lower 1-, 5-, and 10-year survival rates of 87%, 55%, and 36% than patients without vascular invasion (91%, 78%, and 65%, *p* < 0.001). The overall HCC recurrence rate was 12%, 24%, and 30% after 1, 5, and 10 years. Patients with microvascular invasion revealed recurrence rates of 26%, 48%, and 53% after 1, 5, and 10 years, which was significantly higher than without microvascular invasion (4%, 10%, and 16%; *p* < 0.001). Characteristics of the groups with and without microvascular invasion are given in Table 1. Size and number of tumor nodules were significantly higher in patients with vascular invasion as was the mean serum AFP level (Table 1).

**Figure 1:**
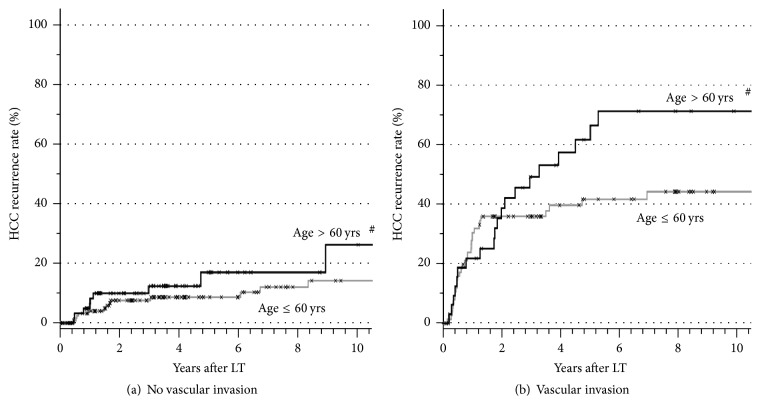
HCC recurrence rates in patients without (a) and with (b) microvascular invasion in correlation with the recipient's age at transplantation (^#^ *p* < 0.05 age ≤ 60 versus age > 60 tested by stratified log rank test).

**Figure 2:**
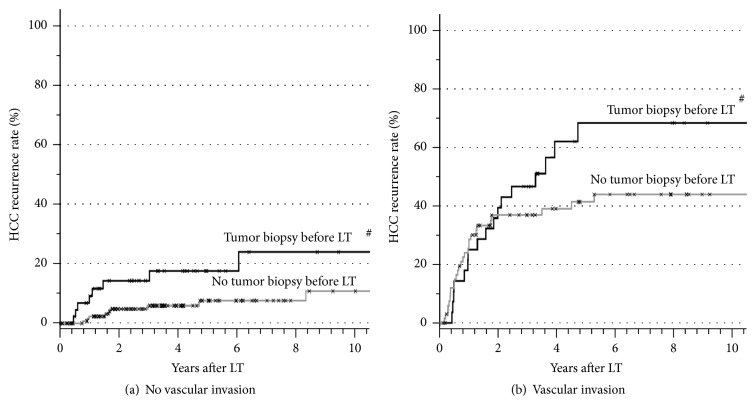
HCC recurrence rates in patients without (a) and with (b) microvascular invasion in correlation with pretransplant tumor biopsy (^#^ *p* = 0.015 for tumor biopsy versus no tumor biopsy tested by stratified log rank test).

**Figure 3:**
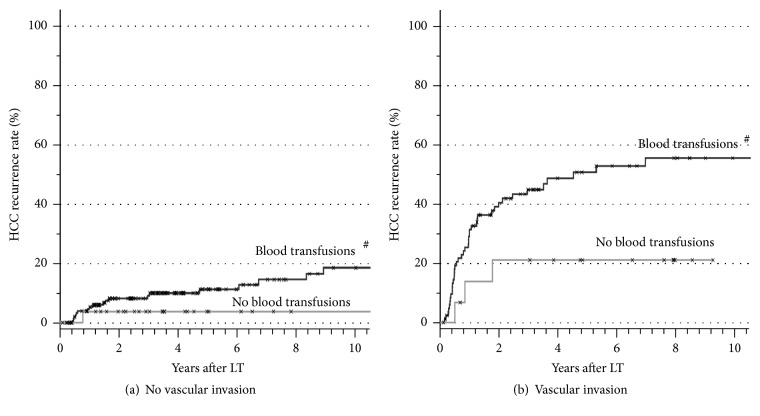
HCC recurrence rates in patients without (a) and with (b) microvascular invasion in correlation with application of blood transfusions intra- or postoperatively (^#^ *p* = 0.023 for blood transfusions versus no blood transfusions tested by stratified log rank test).

**Figure 4:**
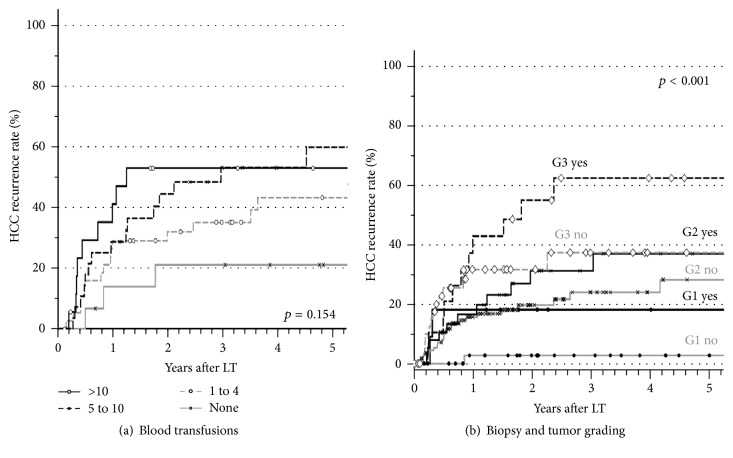
Actuarial HCC recurrence (a) in patients with microvascular invasion in correlation with the number of applied blood transfusions (*p* = not significant) and (b) HCC recurrence rates in patients with (yes) and without (no) tumor biopsy stratified for tumor grading (*p* < 0.001 for biopsy yes versus no stratified for tumor grading G1 to G3).

The *p* values of all analyzed factors for HCC recurrence after LT are given in Table 2. The univariate analysis stratified for microvascular invasion revealed age over 60 years at LT (Figure 1), pretransplant tumor biopsy (Figure 2), and the application of blood transfusions (Figures 3 and 4(a)) as significant risk factors for tumor recurrence. However, only blood transfusions remained significant in the multivariate analysis (Table 2). The application of blood transfusions (yes/no) correlated with tumor recurrence and patient survival. The effect was more pronounced in patients with vascular invasion (Figure 3). In case of blood transfusions, recurrence occurred earlier in patients with more transfusions; however this did not reach statistical significance (Figure 4(a)).

Other risk factors like immunosuppression or rejection treatment revealed no significant influence. Thus, neither the type of calcineurin inhibitor nor the number of combined immunosuppressive agents or the use of antilymphocyte antibodies as induction or rejection treatment influenced the HCC recurrence rate significantly.

To examine a possible selection bias for patients with tumor biopsy before transplantation several subgroup analyses were added. For example, since tumor biopsy was often not considered necessary in patients with high AFP levels, a subgroup analysis of patients with normal AFP levels with and without tumor biopsy was performed. In this subgroup of patients a significantly increased recurrence rate was seen after tumor biopsy (*p* < 0.05 by univariate analysis). The risk of HCC recurrence after previous tumor biopsy was increased in each category of tumor grading by 10% to 20% (Figure 4(b)).

**Figure 5:**
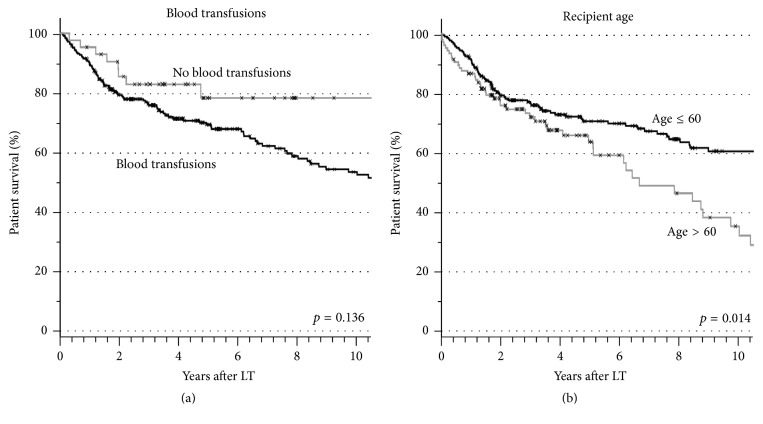
Actuarial patient survival in correlation with the application of blood transfusions (a) and with recipient's age (b).

The patient survival was also influenced by nontumor related characteristics, but the factors tumor biopsy as well as application of blood transfusions failed to reach statistical significance (Figure 5). The only nontumor related factor with significant influence on overall patient survival was age >60 years at transplantation.

## 4. Discussion

It is well known that primary tumor characteristics determine the risk of HCC recurrence after LT [2, 6]. This retrospective analysis has been performed to unravel additional potential risk factors. To compensate for different tumor characteristics, patients were stratified for vascular invasion.

Postoperative complications and administration of blood transfusions are known to impair the immune-competence and therefore might facilitate tumor recurrence following liver resection [13]. Interestingly, the choice of immunosuppressive drugs did not influence tumor recurrence in our analysis and even not the application of ATG. A recent analysis found similar results for OKT 3 [14]. Sirolimus therapy, which has been shown to improve the survival after HCC recurrence in several reports [15, 16], did not significantly lower the recurrence rate in our analysis. However, sirolimus was used predominantly in patients with more advanced tumors, making a selection bias possible.

However, application of blood transfusions during the primary hospital stay was strongly associated with tumor recurrence over the whole study period. This factor has been subject of controversy in several studies after liver resection but has not been addressed after LT. In experimental and clinical studies, hemorrhage can lead to a long-lasting depression of specific and nonspecific immunity [17]. High amounts of allogeneic blood transfusions are known to increase the risk of postoperative complications such as infections and pulmonary complications, which subsequently result in a poorer outcome [18]. In addition, it has been postulated that transfusion-induced immunosuppression may cause harm to patients with cancer [19]. Accordingly, several studies have shown that allogeneic blood transfusions can promote the recurrence of colorectal [20], lung [21], and gastric cancer [22]. However, converse results have been published as well [23]. Likewise, the association between HCC recurrence and blood transfusions after liver resection has been supported [10, 24] and disputed [25]. In the event of liver resection for HCC, several immunosuppressive effects linked to transfusions have been observed. Specifically the NK cell activity, which plays a role in the first-line defense against tumor growth, was found to be decreased at days 7 and 28 in transfused patients [26]. After liver resection an association of blood transfusions with survival might be confounded by individual factors as extent of surgery and postoperative liver function. These confounding variables are less pronounced during LT, where all patients receive the same operation. In our series HCC recurrence and overall survival rate were independently influenced by application of blood products. Transfusion-related immunosuppression in combination with immunosuppressive drug application might hinder the clearance of occult disseminated tumor cells, since these can be detected in blood samples in most patients outside the Milan criteria [27]. From the present data, the pure application of blood transfusions (yes or no) seems to be of relevance. In case of transfusions, a higher number of transfusions do not significantly increase the risk of tumor recurrence. The potential requirement of intraoperative blood transfusions during LT is based on multiple factors, for example, previous abdominal surgery, severity of portal hypertension, and quality of the liver graft. From the present data, one cannot completely exclude that other factors beyond the application of blood transfusions might be relevant for the observed phenomenon. The net state of “immunosuppression” is influenced by numerous factors including drugs, antibodies, transfusions, infections, malnutrition, and many other factors. Therefore possible confounders cannot be ruled out completely in the present analysis.

HCCs seem to be particularly prone to tumor seeding after biopsy with an incidence of 2% to 5% [28, 29]. In comparison, other tumor entities such as pancreatic tumors display needle seeding probabilities below 0.1% following biopsy [28–30], Tumor cell seeding has also been described after LT [31–33], but its impact on outcome after LT has never been analyzed systematically. Whereas in the nontransplant setting needle tract implantation is mainly addressed [34, 35], tumor cell dissemination might be eased as well. In an animal model fine needle aspiration induced implantation of 1000 to 100,000 cells along the needle tract [36]. Dissemination of cells into the tract additionally results in extravasation of tumor cells into blood and lymphatic vessels and might cause distant metastases [37]. An increased risk for tumor recurrence could be shown after percutaneous biopsy in the present study could be shown, although these findings were not confirmed statistically in the multivariate analysis, presumably due to small patient numbers. In a recent review of the literature the risk of seeding after HCC biopsy in nontransplant patients was likewise found to be substantial, especially after diagnostic puncture when compared to therapeutic puncture [29]. This risk is particularly relevant before LT, as shown in the present analysis. Nowadays the sensitivity and specificity of noninvasive imaging studies in HCC suspicious lesions larger than 1 cm are both higher than 90% [38] and therefore biopsy is not routinely necessary [39, 40]. However, more than 20% of patients underwent tumor biopsy in the present series, even in more recent years. This is due to the fact that the majority of biopsies were already performed before referral to the transplant center. Therefor the frequency of biopsies does not reflect the centers policy.

Furthermore, in the present analysis, recipient age was found to be another factor influencing recurrence rates of HCC after LT. It is well known that older HCC patients have lower survival rates following LT [41]. In our analysis the HCC recurrence rate was also increased in patients over 60 years. However, the reason remains unclear.

In the present large series, three nontumor related risk factors for HCC recurrence after LT were identified. Our findings highlight the importance of minimizing operative blood loss for oncological reasons. Additionally, tumor biopsy before LT might increase the risk of HCC recurrence and therefore its risks have to be individually weighed against potential benefits. The relevance of other less dominant factors could not be statistically proven in the present analysis. However, due to the retrospective nature of this study and the relatively long study period with slight modifications in immunosuppression and perioperative management, the relevance of other confounding factors cannot be completely excluded.

## Figures and Tables

**Table 1:** Comparison of patient and tumor characteristics in the subgroups with and without microvascular invasion.

	No vascular invasion (*n* = 222)	Vascular invasion (*n* = 114)	*p* =
Male	174 (78%)	100 (88%)	0.037

Mean age at LT [years]	57 ± 1	56 ± 1	0.840

Era of transplantation			0.011
(i) Before 1997	47 (21%)	29 (25%)	
(ii) 1997–2003	58 (26%)	44 (39%)	
(iii) 2004 to 2008	117 (53%)	41 (36%)	

Underlying liver disease			0.777
(i) Alcohol	57 (26%)	34 (30%)	
(ii) HCV	81 (37%)	39 (34%)	
(iii) HBV	36 (16%)	19 (17%)	
(iv) Cryptogenic	35 (16%)	14 (12%)	
(v) Others	13 (6%)	8 (7%)	

Recipient blood group			0.958
(i) 0	58 (29%)	33 (30%)	
(ii) A	110 (55%)	58 (52%)	
(iii) B	22 (11%)	14 (13%)	

Mean waiting time	142 ± 13	124 ± 15	0.120

Child Pugh stadium			0.809
(i) A	95 (43%)	44 (39%)	
(ii) B	97 (44%)	51 (45%)	
(iii) C	30 (14%)	19 (17%)	

TACE before LT	60 (27%)	29 (25%)	0.888

Previous liver resection	23 (10%)	6 (5%)	0.163

Piggy back	71 (32%)	27 (24%)	0.263

Tumor biopsy			0.553
(i) Yes	46 (21%)	29 (25%)	
(ii) No	147 (66%)	69 (61%)	
(iii) Unknown	29 (13%)	16 (14%)	

No blood transfusions (24 h)	28 (15%)	15 (15%)	0.561

Mechanical ventilation >24 h	11 (6%)	9 (9%)	0.255

Haemodialysis after LT	27 (12%)	16 (14%)	0.496

Number of tumor nodules			0.055
(i) ≤3	195 (88%)	91 (80%)	
(ii) >3	27 (12%)	23 (20%)	

Maximum tumor diameter			<0.001
(i) ≤5 cm	194 (87%)	79 (69%)	
(ii) >5 cm	28 (13%)	35 (31%)	

Mean max. diameter [cm]	3.1 ± 0.2	4.7 ± 0.3	0.005

Mean AFP [*μ*g/l]	583 ± 241	3120 ± 1350	0.005

AFP category			0.023
(i) <20	115 (52%)	46 (40%)	
(ii) 21–100	45 (20%)	15 (13%)	
(iii) 101–1000	33 (15%)	25 (22%)	
(iv) >1000	12 (5%)	14 (12%)	
(v) Not determined	17 (8%)	14 (12%)	

Tumor grading			<0.001
(i) G1	54 (24%)	7 (6%)	
(ii) G2	121 (55%)	60 (53%)	
(iii) G3	27 (12%)	13 (40%)	
(iv) Gx	20 (9%)	1 (1%)	

**Table 2:** Univariate and multivariate *p* values of tumor and nontumor related risk factors for HCC recurrence after LT. The nontumor related risk factors were analyzed stratified for the most dominant tumor related risk factor (microvascular invasion).

	Univariate	Multivariate
Tumor factors:		
Microvascular invasion	**<0.001**	**<0.001**
Number of tumor nodules (≤3 versus >3)	**<0.001**	0.138
Maximum diameter (≤5 versus >5 cm)	**0.001**	0.934
AFP level	**0.006**	0.495
HCC incidentally detected after LT	0.108	

Nontumor factors:		
Tumor biopsy	**0.015**	0.884
Blood transfusions	**0.023**	**0.033**
Age (≤60 versus >60 years)	**0.045**	0.118
Waiting time	0.091	
Child Pugh status	0.175	
Sirolimus after LT	0.183	
Underlying liver disease	0.208	
Cyclosporine (versus tacrolimus)	0.280	
Haemodialysis	0.343	
Immunosupp. (dual versus triple versus quadruple)	0.362	
ATG induction therapy	0.398	
Pervious liver resection for HCC	0.481	
Gender	0.496	
OKT 3 therapy	0.524	
Piggy back	0.567	
Azathioprine	0.571	
IL-2 induction therapy	0.614	
Antirejection therapy (antibodies, steroids)	0.646	
Mycophenolate	0.674	
Duration of ICU treatment	0.746	
Period of LT	0.859	
Blood group	0.891	
TACE before LT	0.960	

## Abbreviations

AFP: Alpha-fetoprotein
HCC: Hepatocellular carcinoma
LT: Liver transplantation
TACE: Transarterial chemoembolization.
